# Bioactive (Poly)phenols, Volatile Compounds from Vegetables, Medicinal and Aromatic Plants

**DOI:** 10.3390/foods10010106

**Published:** 2021-01-06

**Authors:** Teresa Pinto, Alfredo Aires, Fernanda Cosme, Eunice Bacelar, Maria Cristina Morais, Ivo Oliveira, Jorge Ferreira-Cardoso, Rosário Anjos, Alice Vilela, Berta Gonçalves

**Affiliations:** 1 CITAB, Centre for the Research and Technology of Agro-Environmental and Biological Sciences, Department of Biology and Environment, School of Life Sciences and Environment, University of Trás-os-Montes and Alto Douro, P-5000-801 Vila Real, Portugal; areale@utad.pt (E.B.); ivo.vaz.oliveira@utad.pt (I.O.); jventura@utad.pt (J.F.-C.); ranjos@utad.pt (R.A.); bertag@utad.pt (B.G.); 2 CITAB, Centre for the Research and Technology of Agro-Environmental and Biological Sciences, University of Trás-os-Montes and Alto Douro, P-5000-801 Vila Real, Portugal; alfredoa@utad.pt (A.A.); cmorais@utad.pt (M.C.M.); 3 CQ-VR, Chemistry Research Centre, Department of Biology and Environment, School of Life Sciences and Environment, University of Trás-os-Montes and Alto Douro, P-5000-801 Vila Real, Portugal; fcosme@utad.pt (F.C.); avimoura@utad.pt (A.V.)

**Keywords:** aromatic plants, bioactive compounds, consumers, medicinal plants, phenolic compounds, plant breeders, volatile compounds, vegetables

## Abstract

Polyphenols, as well as volatile compounds responsible for aromatic features, play a critical role in the quality of vegetables and medicinal, and aromatic plants (MAPs). The research conducted in recent years has shown that these plants contain biologically active compounds, mainly polyphenols, that relate to the prevention of inflammatory processes, neurodegenerative diseases, cancers, and cardiovascular disorders as well as to antimicrobial, antioxidant, and antiparasitic properties. Throughout the years, many researchers have deeply studied polyphenols and volatile compounds in medicinal and aromatic plants, particularly those associated with consumer’s choices or with their beneficial properties. In this context, the purpose of this review is to provide an overview of the presence of volatile and nonvolatile compounds in some of the most economically relevant and consumed vegetables and medicinal and aromatic plants, with an emphasis on bioactive polyphenols, polyphenols as prebiotics, and, also, the most important factors that affect the contents and profiles of the volatile and nonvolatile compounds responsible for the aromatic features of vegetables and MAPs. Additionally, the new challenges for science in terms of improving polyphenol composition and intensifying volatile compounds responsible for the positive characteristics of vegetables and medicinal and aromatic plants are reported.

## 1. Introduction

The concept of “quality” is wide, but in horticulture, it can be defined as the degree of excellence given by the combination of different attributes or characteristics that give each product value in terms of its proposed use [1]. In this concept, visual appearance, ability to endure postharvest processing operations, chemical and nutritional composition, and aroma can be included [2]. Advances have been made in horticultural breeding, and now it is possible to find fruits and vegetables with characteristics that growers and retailers desire, such as high yield, high resistance to pest attacks and disease, attractive appearance, and capacity to support different handling and processing operations. However, most of the time, many of these horticultural crops fail to achieve top nutritional and flavour characteristics [3]. Increasing horticultural crops’ flavour by breeding is still not an easy task, due to the multitude of factors that affect the synthesis of volatile and nonvolatile compounds responsible for flavour attributes such as climate, cultural practices, agricultural practices (organic vs. conventional), and pre- and postharvest processing operations [4]. Additionally, the astringency, dryness, viscosity, heat, coolness, prickling, and pain, often referred to as the “texture” of foods, can affect the flavour of vegetables and medicinal and aromatic plants (MAPs) [5]. This review presents a discussion of the most important factors that affect the contents and profiles of the volatile and nonvolatile compounds responsible for the aromatic features of vegetables and MAPs, as well as the recent advances in plant breeding regarding the achievement of chemical compounds responsible for the typical aromatic features’ sensory attributes.

## 2. Plant Bioactive Phenolic Compounds

Vegetables and MAPs are important sources of bioactive phenolic compounds and have a key role in the development of compounds eliciting beneficial health effects [6]. Phenolic bioactive compounds of plant origin are those secondary metabolites possessing desired health benefit effects [7]. They might be produced from two distinct pathways: (i) shikimic acid (phenylpropanoids) and (ii) acetic acid (phenols) [8]. Due to their abundance in vegetables and MAPs, the study of phenolic compounds’ (simple phenolics, coumarins, lignans, flavonoids, isoflavonoids, anthocyanins, proanthocyanidins, and stilbenes) effects on health has increased in recent years, due to the growing evidence indicating that polyphenols are a major class of bioactive phytochemicals. Their consumption may play a role in the prevention of several chronic diseases as potent antioxidant properties, prevention of diseases induced by oxidative stress, and prevention of some specific cardiovascular (mainly high cholesterol levels, high blood pressure) and neurodegenerative diseases (such as Alzheimer’s or Parkinson’s, type II diabetes, cancers, urinary tract infections) [9,10,11,12,13]. However, the health effects of phenolic compounds are dependent on their type, quantity consumed, as well as on their bioavailability.

The amount of total phenolic compounds is greater in dark vegetables, such as red kidney beans, black beans (*Phaseolus vulgaris*), and black gram (*Vigna mango*). Bravo [9] determined (by regarding dry matter, mg/100 g) the amount of total phenolic compounds in several vegetables such as black gram (540–1200), chickpeas (78–230), cowpea (175–590), common beans (34–280), green gram (440–800), pigeon peas (380–1710), Brussel sprouts (6–15), cabbage (25), leek (20–40), onion (100–2025), parsley (55–180), and celery (94).

**Figure 1:**
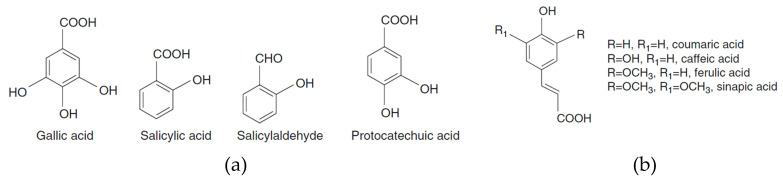
Chemical structures of hydroxybenzoic acids (**a**) and hydroxycinnamic acids (**b**).

Phenolic acids have been recently widely studied because of their potential protective roles. Phenolic acids have a benzene ring, a carboxylic group, and one or more hydroxyl and/or methoxyl groups. They are usually divided into benzoic acid derivatives (i.e., hydroxybenzoic acids, C_6_-C_1_) (Figure 1a) and cinnamic acid derivatives (i.e., hydroxycinnamic acids, C_6_-C_3_) (Figure 1b), based on the constitutive carbon structures. The amount of hydroxybenzoic acid (C_6_-C_1_ derivatives) (e.g., gallic acid, salicylic acid, salicylaldehyde, and protocatechuic acid) is typically low in edible plants [14]. Phenolic acids may make up about one-third of the phenolic compounds in the human diet; these substances have a powerful antioxidant activity that may help protect the body from free radicals [9,15].

According to Khadem and Marles [16], gallic acid has antineoplastic and bacteriostatic activities, and salicylic acid exerts anti-inflammatory, analgesic, antipyretic, antifungal, and antiseptic properties. Protocatechuic acid has also been described as having several bioactivities such as anti-inflammatory, antifungal, and antioxidant ones [17]. For instance, *p*-hydroxybenzoic acid has been isolated from many sources including carrots (*Daucus carota*) [18] and protocatechuic acid from onion, garlic, and relatives (*Allium* spp.) [19].

The hydroxycinnamic acids (C_6_-C_3_ derivatives) are more abundant than the hydroxybenzoic acids. The four most common hydroxycinnamic acids are ferulic acid, caffeic acid, coumaric acid, and sinapic acid. These acids are frequently present in plants in the combined forms such as glycosylated derivatives or esters of tartaric acid, shikimic acid, and quinic acid rather than in the free form. Hydroxycinnamic acids are recognized as powerful antioxidants playing an essential role in protecting the body from free radicals. Several hydroxycinnamic acid derivatives, such as caffeic acid, chlorogenic acid, ferulic acid, *p*-coumaric acid, and sinapic acid, present strong antioxidant activities by inhibiting lipid oxidation and scavenging reactive oxygen species (ROS) [10]. Chlorogenic acid and caffeic acid inhibit the N-nitrosation reaction and prevent the formation of mutagenic and carcinogenic N-nitroso compounds [20].

**Figure 2:**
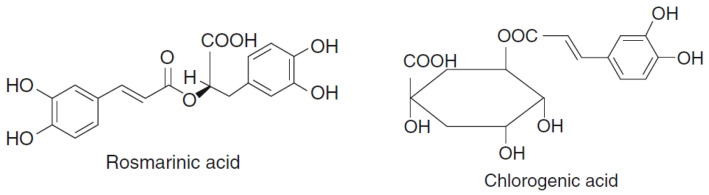
Chemical structures of rosmarinic acid and chlorogenic acid.

Rosemary (*Rosmarinus officinalis* L.) extracts have been used as diuretic, analgesic, expectorant, antirheumatic, and antimutagenic agents. Caffeic acid and its derivatives, such as rosmarinic acid (Figure 2) and chlorogenic acid, have been thought to be the most important ones responsible for the therapeutic properties of rosemary extracts, as they have antioxidant effects and contribute to the bioactive function of rosemary [21].

Among the phenolic compounds identified by Zheng and Wang [22], rosmarinic acid was the predominant phenolic compound in *Salvia officinalis* and *Thymus vulgaris* (Table 1).

In previous years, the use of the active phenolic acid compounds (such as chlorogenic acid, ferulic acid, cinnamic acid, and rosmarinic acid) in food has increased. Thus, the study of plants’ phytochemicals is important and essential [23].

Coumarins are a large class of C_6_-C_3_ derivatives belonging to the benzo-α-pyrone group, which exist in the free or combined form as heterosides and glycosides in certain plants; most of them are isolated from chlorophyll-containing plant materials [24]. Species rich in coumarins included *Aesculus hippocastanum* (Horsechestnut), *Passiflora incarnata* (Passionflower), *Lawsonia inermis* (Henna), *Hypericum perforatum* (Saint John Wort), *Tilia cordata* (Lime Tree), and *Uncaria tomentosa* (Cat’s Claw) [25]. Coumarins can be categorised into four types. Simple coumarins are the hydroxylated, alkoxylated, and alkylated derivatives of the benzene ring of coumarin, and the corresponding glycosides. Furanocoumarins compounds consist of a five-member furan ring attached to the coumarin nucleus, divided into linear and angular types with a substituent at one or both remaining benzenoid positions. Pyrano coumarins are analogous to the furanocoumarins but contain a six-member ring. The last type is coumarins substituted in the pyrone ring [26]. Several products that contain a coumarin moiety show excellent biological activities such as antitumor, antibacterial, antifungal, anticoagulant, vasodilator, analgesic, and anti-inflammatory activities [24,27,28].

Lignans are a diverse group of bioactive phenolic compounds formed of two β-β-linked phenylpropane units; they are present in different parts of plant species in free form or combined form as glycoside derivatives. Lignans are found in vegetables such as in the brassica family where fresh edible weights (mg/100 g) between 0.185 to 2.32 of can be found, for instance, for broccoli (98.51), Brussels sprouts (50.36), cauliflower (9.48), green cabbage (0.03), red cabbage (18.1), white cabbage (21.51), and kale (63). They can also be found in green beans (22.67), tomato (2.15), cucumber (3.8), zucchini (7.02), green lettuce (1.17), and carrot (7.66). However, spinach, white potatoes, and mushrooms contain an amount below 0.1 mg/100 g (fresh edible weight) of lignin [29,30]. Lignan presents a great antioxidant activity and may be effective in the treatment of cardiovascular disease, coronary heart disease, and diabetes [31].

Flavonoids have the general structural C_6_-C_3_-C_6_, in which the two C_6_ units are phenolic and linked by a C_3_ group. They can be divided into flavones, flavonols, flavanones, and flavanols, according to the oxidation state of the central pyran ring, as well as in anthocyanins and isoflavonoids, with different antioxidant, antibacterial, antiviral, and anticancer activities [15,32].

**Figure 3:**
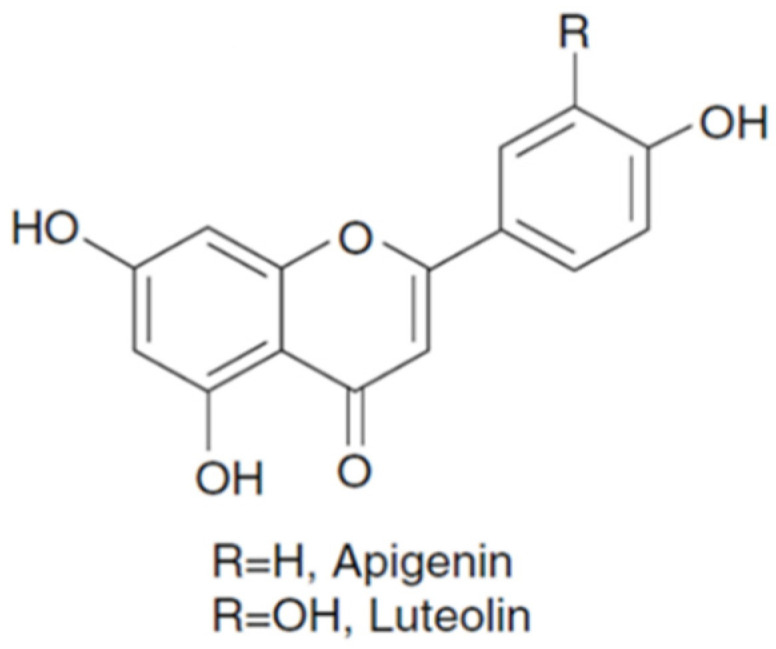
Structures of the major flavones.

Flavones usually occur as glycosides of apigenin and luteolin in plants (Figure 3). Flavones are found in celery (22–108 mg/kg fresh weight) and showed the proprieties of lowering the levels of total and low-density lipoprotein (LDL) cholesterol and also have anti-inflammatory and anticancer activities [33]. In other vegetables, the amounts are (mg/kg of luteolin and apigenin, respectively): 0.41 and 0.05 in water spinach; 0.09 and 0.03 in cucumber; 0.16 and 1.07 in purple cabbage; 1.18 and 0.31 in Chinese cabbage; 0.16 and 0.92 in white cabbage and 0.22 and 0.04 in onion [34].

**Figure 4:**
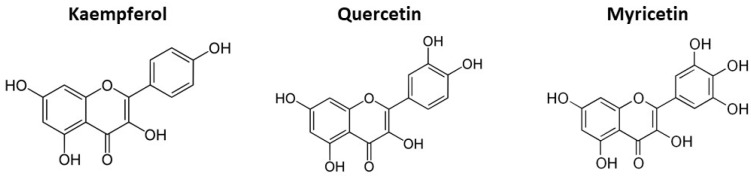
Structures of the major flavonol aglycones.

Flavonols have been extensively studied and are extensively distributed in plants [35,36,37,38,39,40,41,42,43,44]. They are frequently the conjugated form of glycosides such as kaempferol, quercetin, and myricetin (Figure 4). Quercetin levels in the edible parts of most vegetables are generally (of fresh weight, mg/kg) below 10, except for onions (284–486), kale (110), broccoli (30), French beans (32–45), and slicing beans (28–30) [35]. Kaempferol could only be detected (fresh edible weight, mg/kg) in kale (211), endive (15–91), leek (11–56), and turnip tops (31–64) [35]. A rich source of flavonols are onion leaves that contain (fresh weight, mg/kg) 1.497 of quercetin and 832 of kaempferol [37], and also sweet potato leaves (purple) showed 156 mg/kg of myricetin and 267 mg/kg of quercetin [34]. According to Erlund [45], quercetin is an antioxidant protecting against reactive oxygen species and shows also antiatherosclerosis, anticancer, anti-inflammatory, and cholesterol-lowering properties. Flavanones are colourless compounds characterised by the absence of a double bond in the 2, 3-position of the pyrone ring, and are isomeric with chalcones. Low concentrations of flavanones, namely naringenin, are found in tomatoes [46].

**Figure 5:**
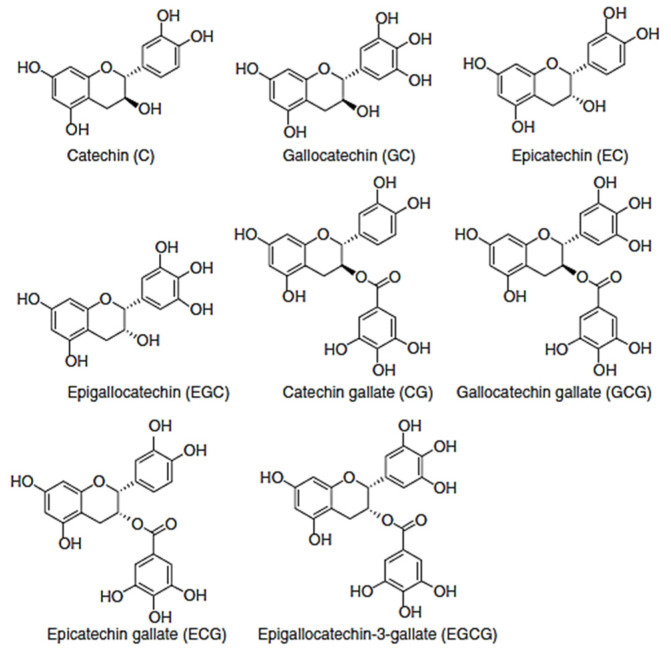
Structures of monomeric flavan-3-ols.

Monomeric flavan-3-ols include catechin, epicatechin, gallocatechin, catechin gallate, epicatechin gallate, epigallocatechin, epigallocatechin-3-gallate, and gallocatechin gallate (Figure 5). Catechin and epicatechin are the most abundant flavanols found in fruits, while in the seeds of some leguminous, the most abundant flavanols are gallocatechin, epigallocatechin, and epigallocatechin gallate [47]. In fava beans (*Vicia faba* L.), (−)-epicatechin and epigallocatechin were detected by Helsper et al. [48]. A general trend of increasing “total catechin equivalent” content with increasing darkness of the legumes within one family can be observed [49,50]. All types of beans, and mature seeds contain flavan-3-ols (mg/100 g, edible portion)—namely, (+)-catechin (1.66); (−)-epicatechin (0.35) [51] and beans, pinto, mature seeds, raw (*Phaseolus vulgaris*) (+)-catechin (5.07); (−)-epicatechin (0.14); (−)-epigallocatechin (0.05 mg/100 g) [52], broad beans, immature seeds, raw (*Vicia faba*), (−)-epicatechin (28.96); (−)-epigallocatechin (15.47); (+)-catechin (14.29); (+)-gallocatechin (4.15) [51,52]. Catechin prevents protein oxidation by its free radical scavenging capacity. Furthermore, it possesses the ability to reduce the covalent modification of protein induced by reactive oxygen species (ROS) or by-products of oxidative stress [53].

**Figure 6:**
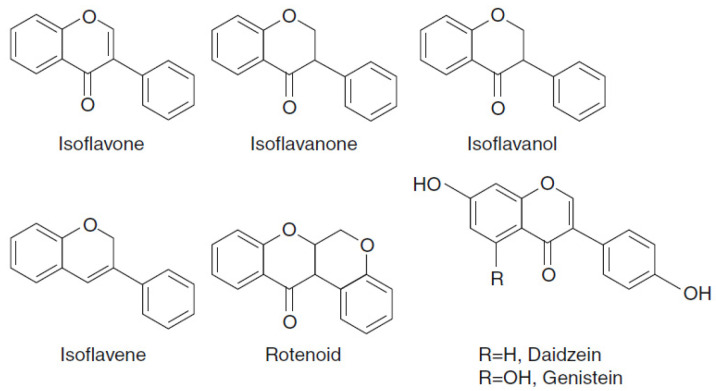
Chemical structures of isoflavonoids.

Isoflavonoids are flavonoids that have their B ring fused with the C_3_ position of ring C, which are phenolics with phytoestrogenic activity (Figure 6). The concentrations of isoflavones in soybean products ranged from 580 to 3800 mg/kg of fresh weight [54].

**Figure 7:**
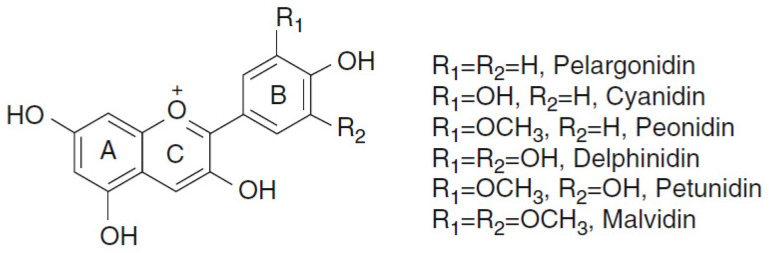
Chemical structures of anthocyanidins.

The basic structures of anthocyanins are anthocyanidins, in which the two aromatic rings A and B are linked by a heterocyclic ring C that possesses oxygen. More than 23 different anthocyanidins have been found with pelargonidin, cyanidin, peonidin, delphinidin, petunidin, and malvidin being the most common (Figure 7). Anthocyanins in plants mainly exist in conjugated form as glycosides. Monomeric anthocyanin changed the hydroxylation and methoxylation patterns on the B ring; the nature, position, and the number of conjugated sugar units; the nature and number of conjugated aliphatic or aromatic acid groups; the existence or lack of an acyl aromatic group in the molecule [55]. They are usually present in any pink to purple vegetables such as black beans (*Phaseolus vulgaris*) (delphinidin (11.98); malvidin (6.45); petunidin (9.57) in mg/100 g, edible portion); kidney red beans (*Phaseolus vulgaris*) (pelargonidin (2.42); cyanidin (1.19) in mg/100 g, edible portion) [56]; common raw beans (*Phaseolus vulgaris* var. Zolfino) (delphinidin (2.50); malvidin (0.10); petunidin (0.14) in mg/100 g, edible portion) [38]; redraw cabbage (*Brassica oleracea*) (cyanidin (72.86), delphinidin (0.01); pelargonidin (0.02) in mg/100 g, edible portion) [42,56] and in cowpeas (blackeyes, crowder, southern) (*Vigna unguiculata*) (cyanidin (94.72); delphinidin (94.60); malvidin (34.28); peonidin (11.07); petunidin (27.82) in mg/100 g, edible portion) [57]. The protective effects of anthocyanins include antiedema, antioxidant, anti-inflammatory, and anticarcinogenic activities [58].

**Figure 8:**
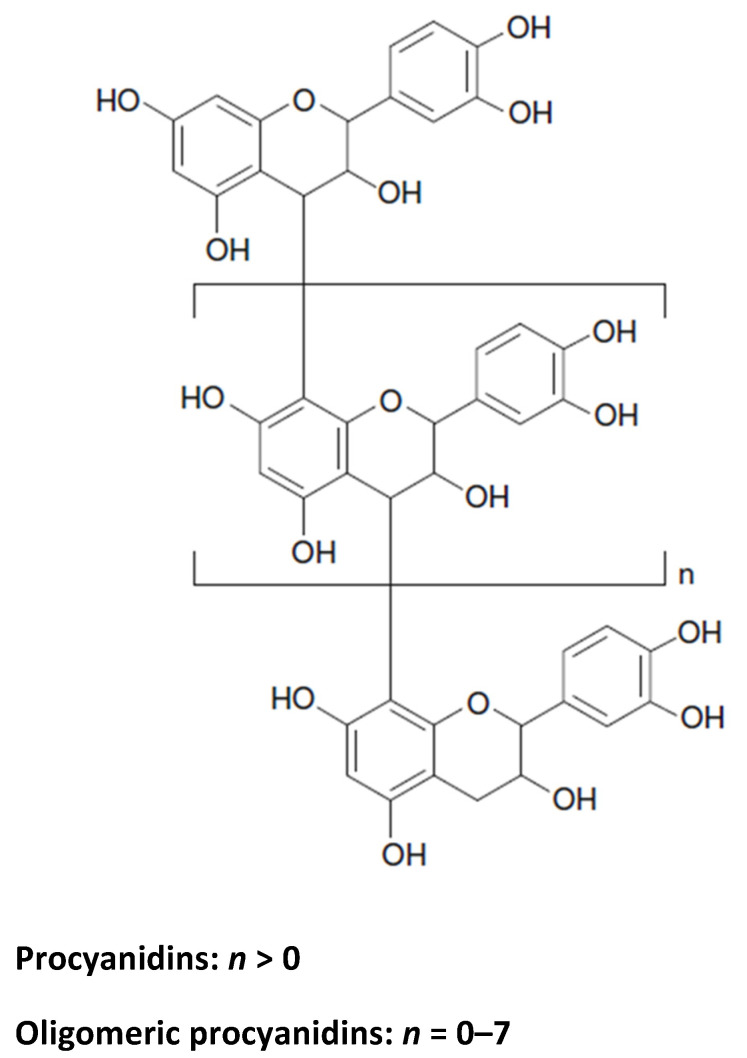
Chemical structure of procyanidins.

Condensed tannins, also recognised as proanthocyanidins, mainly comprise a flavan-3-ol unit to form dimers, oligomers, and polymers of up to 50 monomer units (Figure 8). Proanthocyanidins have complex structures depending on the number of the flavan-3-ol units, the location and type of interflavan linkage in the molecule, and the nature and position of substituents on the flavan-3-ol unit. Proanthocyanidins can be classified into procyanidins and prodelphinidins based on their hydroxylation patterns of A and B rings [33]. The proanthocyanidin contents in spinach (*Spinacea oleracea*) and radish leaves (*Raphanus sativus*) are 88.46 and 13.57 proanthocyanidins in mg/100 g fresh weight, respectively [59]. Proanthocyanidins have antioxidant activity responsible for cardioprotection, cancer chemoprevention, and lowering cholesterol amounts [33].

**Figure 9:**
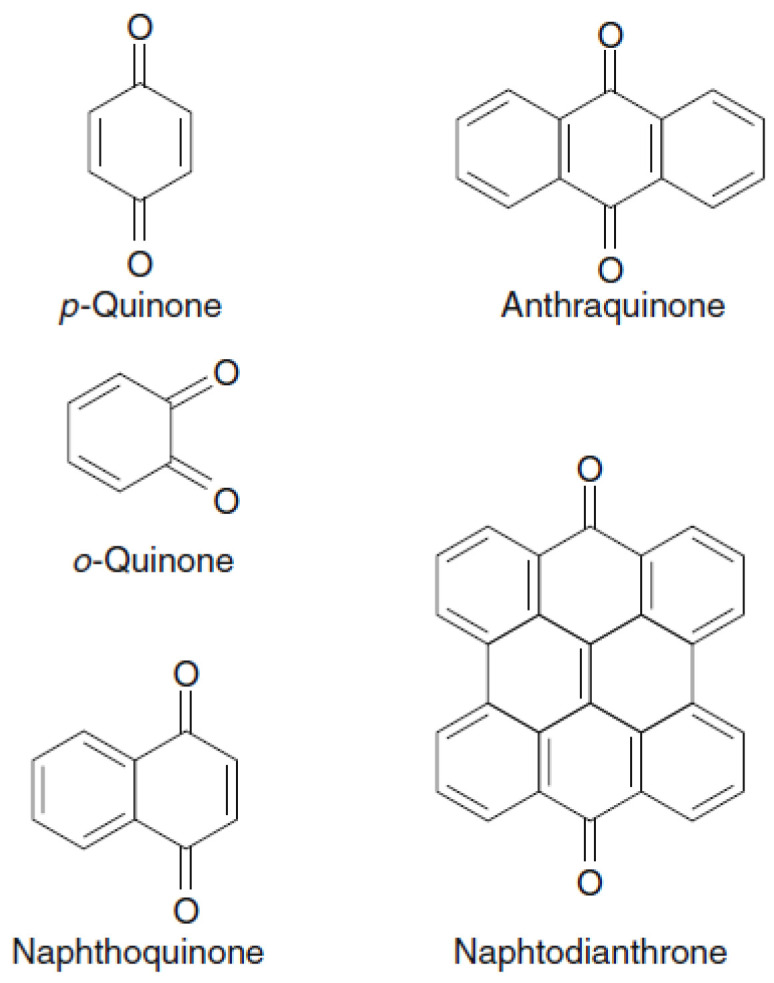
Quinone structures.

Quinones are phenolic compounds with conjugated cyclic dione structures, such as that of benzoquinones, derived from aroma compounds by the conversion of an even number of –CH= groups into –C(=O)– groups with any necessary rearrangement of double bonds. The most common skeletal structures of quinones found in plants are *p*-quinone, *o*-quinone, anthraquinone, naphthoquinone, and naphtodianthrone (Figure 9).

**Figure 10:**
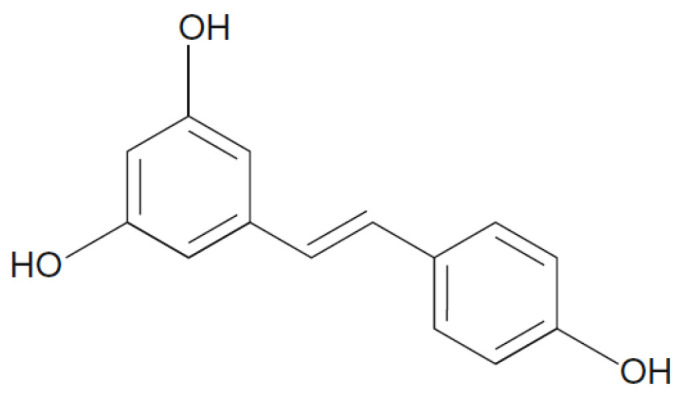
Chemical structure resveratrol.

Stilbenes are a group of phenolic compounds that share a similar chemical structure to flavonoids, in which the two aromatic rings (A and B) are linked by a methylene bridge. One of the most aroma compounds stilbenes is present mostly in glycosylated forms is *trans*-resveratrol (Figure 10). Resveratrol is a phytoalexin that has been particularly studied as it shows several biological activities, reduces the formation of atherosclerotic plaque, present neuroprotective, antidiabetic, anti-inflammatory, antioxidant, anticarcinogenic effects, and antiviral activity [60,61]. It was also shown in several studies that *trans*-piceid a 3-β-glucosylated form of *trans*-resveratrol could inhibit platelet aggregation [62,63] and oxidation of human low-density lipoprotein (LDL). Peng et al. [64] showed that *trans*-piceid was the major form existing in most vegetables, and most of the samples contained higher *trans*-piceid than *trans*-resveratrol. The concentration of *trans*-resveratrol in μg/100 g fresh weight lies between 1.14 and 0.70 in cauliflower and 1.78 and 23.12 in celery, as well as 8.8 and 19.74 in black soya bean. As for *trans*-piceid, it is between 43.04 and 783.29 in celery, 0.80 and 9.22 in leaf lettuce, 1.10 and 12.0 in tomato, and 18.16 and 194.40 in red radish [64]. According to Sebastià et al. [65], the concentration of *trans*-resveratrol in tomatoes is 0.2 µg/g.

Flavonoids are largely distributed in vegetables and they have been studied mainly because of their potential health benefits as antioxidants and chemopreventive agents [48]. However, until now no recommended daily intake of these compounds has been established mainly because the composition data are incomplete, the biological activities are not well determined, and especially because the bioavailability and pharmacokinetic data are inconclusive. Emerging science from some studies suggests that flavonoid-rich diets may lower the risk of some diet-related chronic degenerative diseases [66,67,68] but a few clinical and laboratory reports indicate that very high doses of certain flavonoids may have adverse effects [69,70]. Therefore, it is important to accurately assess flavonoid intakes from the perspectives of both disease prevention and safety [71,72]. The specific action of each phenolic compound from vegetables and medicinal and aromatic plants is not easy to measure since only a small part of it is truly absorbed and, also, it may potentially transform [73]. Numerous dietary phenolic compounds are antioxidants able to quench ROS and toxic free radicals formed from the peroxidation of lipids and, consequently, have anti-inflammatory and antioxidant properties. Flavonoids are recognised as preventing the production of free radicals by chelating iron and copper ions to directly scavenge ROS and toxic free radicals and inhibit lipid peroxidation, which may damage DNA, lipids, and proteins, linked to ageing, atherosclerosis, cancer, inflammation, and neurodegenerative diseases [74].

Many of these reported biological functions have been attributed to free radical scavenging activity and there has been intensive research on the natural antioxidants derived from plants [32,75,76,77]. Hundreds of epidemiological studies have correlated the antioxidant, anticancer, antibacterial, cardioprotective, anti-inflammation, and immune system promoting roles of plants enhanced by phenolic content. Table 2 and Table 3 summarise important bioactivities related to the presence of phenolic identified in vegetables and MAPs widely consumed in the world. For example, Salem et al. [78] found that extracts of artichokes rich in polyphenols were capable of inhibiting the production of histamine, bradykinin, and chemokines. These authors discovered that polyphenols present in extracts were capable of acting synergistically, enhancing their anti-inflammatory potential. Additionally, Sharma et al. [79] observed that extracts of onion were capable of inhibiting the bacterial growth of *Staphylococcus* sp. and *Escherichia coli*, due to the presence of quercetin aglycone, quercetin-4′-*O*-monoglucoside, and quercetin-3,4′-*O*-diglucoside. However, the intensity of the antagonistic effect was dependent on the concentration of each compound in each onion variety assessed. In 2018, Dzotam et al. [80], using extracts of nutmeg rich in 7-trihydroxyflavone, observed an antibacterial activity of such extracts against the multidrug resistant Gram-negative bacteria *Providencia stuartii* and *Escherichia coli*. A recent study showed that *Thymus* extract rich in rosmarinic acid and 3,4-dihydroxybenzoic acid was capable of exhibiting antiradical and antioxidant properties and enhanced gastrointestinal digestion [81].

### 2.1. Polyphenols as Prebiotics

As mentioned previously, polyphenols are natural compounds present in many vegetables and MAPs. In the human body, the majority of polyphenols have poor absorptions and they are retained in the intestine for more time where they can promote beneficial effects, specifically by affecting the gut microbiota [96,97,98]. This leads to a mutual reaction between polyphenolic compounds and gut microbiota. The polyphenols are biotransformed into low-molecular-weight phenolic metabolites by gut microbiota resulting in an increase in polyphenol’s bioavailability, responsible for the health effects derived from the consumption of polyphenol-rich plants, which may differ from the native compound found in the plants [97,98,99,100,101,102]. The properties of polyphenols are dependent on the bioactive metabolites produced when they are metabolised by the microbiota [103]. At the same time, specific polyphenols can modulate the gut microbial composition frequently by the inhibition of pathogenic bacteria and increase the growth of beneficial bacteria resulting in changes of gut microbial composition [104,105,106,107]. Finally, they may act as prebiotic metabolites and enhance the beneficial bacteria. It was demonstrated in animal studies that the consumption of polyphenols, especially catechin, anthocyanins, and proanthocyanidins, increases the abundance of *Lactobacillus*, *Bifidobacterium*, *Akkermansia*, *Roseburia*, and *Faecalibacterium* spp. [108]. Prebiotics were defined in 1995 as “nondigestible food constituents that beneficially act in the host by selectively stimulating the growth and/or activity of one or a limited number of bacterial species, already resident in the colon” [109]. Later, in 2010, prebiotics was defined as “a selectively fermented ingredient that allows specific changes, both in the composition and/or activity in the gastrointestinal microflora, benefits upon host well-being and health” [110]. Bioavailability of polyphenols is influenced by their structural characteristics, mainly by their degree of polymerisation [111,112]—for example, proanthocyanidins are not absorbed by the intestinal mucosa [112], only aglycones and some glucosides can be absorbed [113]. Additionally, the prebiotic effect of each polyphenol can be influenced by the plant source and the characteristic of the chemical structure of the compound, along with the individual differences in gut microbiota compositions [114].

### 2.2. Advances in Phenolic Compounds and Future Research Perspectives

As plant bioactive phenolic compounds have received increasing attention in recent years [115,116], the research concerning their biosynthesis, biological activities, extraction, purification processes, and chemical characterisations are of the utmost interest. New analytical strategies, such as Nuclear magnetic resonance (NMR) and Mass spectrometry (MS), have demonstrated their use in the identification of new molecular structures and characterisation of plant phenolic profiles [117]. Recently, Jacobo-Velázquez et al. [118] focused on most recent advances in plant phenolic research such as the functional characterisation of enzymes involved in the biosynthesis of flavonoids; the evaluation of pre- and postharvest treatments to increase the phenolic concentrations of different plants and the chemical characterisation of the phenolic profiles from different plants, and the evaluation of their bioactivities. Therefore, the development of analytical methods for exploring qualitative or quantitative approaches to analyse these bioactive phenolic compounds, in different plants, is essential. Sample preparation and optimisation of the extraction process (solid–liquid extraction, ultrasound-assisted extractions, microwave-assisted extractions, supercritical fluid extraction) are essential for achieving higher accuracy of results [119,120,121]. According to Swallah et al. [122] it is difficult to choose a universal method for the preparation and extraction of phenolic compounds from different plants, as they have different polarities, molecular structures, concentrations, hydroxyl groups, and several aromatic rings involved. Their analysis can be carried out by using different methods such as spectrophotometry, gas chromatography, liquid chromatography, thin-layer chromatography, capillary electrophoresis, and near-infrared spectroscopy, which are required to develop rapid, sensitive, and reliable methods [123,124]. Another challenge is the analysis of polymeric phenolic compounds, as their polydispersity results in poor resolution and detection, an example of which is proanthocyanidins, which have polydisperse structures for which method development is needed; consequently, characterising the unknown phenolic is one of the main challenges in the research on plant polyphenols [117].

## 3. Plant Volatile Compounds Responsible for Aromatic Features

### 3.1. Vegetable Volatile Compounds

More than 730 flavour compounds have been identified in vegetables [125,126,127,128,129], including some nonvolatile compounds. For example, in tomatoes more than 400 volatile and nonvolatile compounds are known, although only 30 are present in concentrations higher than 1 µL/L, as summarised in different studies [130,131,132]. Nonetheless, lower concentrations of volatile compounds must be considered, because one compound could be lower than 1µL/L but odour active. In pepper, the “sweetness”, “spicy”, “floral”, and “herbal” characteristics are caused by a mixture of volatile compounds—(*Z*)-3-hexenal, 2-heptanone, (*Z*)-2-hexenal, (*E*)-2-hexenal, hexanol, (*Z*)-3-hexanol, (*E*)-2-hexenol, and linalool and nonvolatile compounds (fructose and glucose) [133]. In vegetables, the presence of flavour and nonflavour compounds are diverse, but the key volatile compounds related to the typical sensory properties of vegetables and their respective aromatic features are summarised in Table 4.

In Table 5 are some examples of compounds responsible for typical sensory attributes found in vegetables.

**Figure 11:**
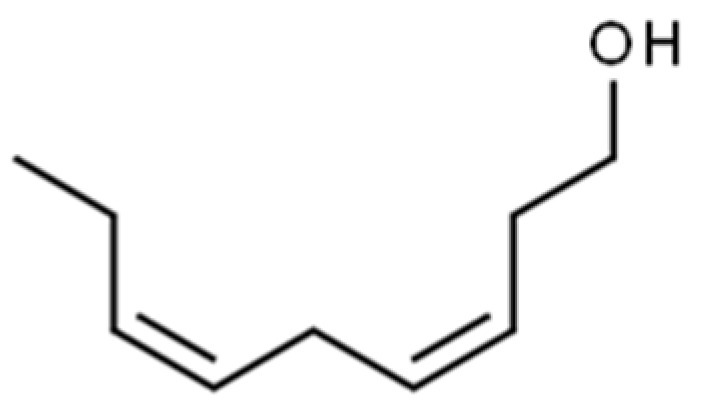
Chemical structure of (*Z*, *Z*)-3,6-nonadienol.

Branched-chain alcohols, which are a result of amino acid deamination and decarboxylation [139,149], are common in plant materials. (*Z, Z*)-3,6-Nonadienol (Figure 11) has been described as having “fatty”, “soapy”, “cucumber”, “watermelon”, and “rind” sensory attributes, in watermelon, but also “boiled leaf-like” and “grassy” attributes in fresh-cut melon [150] or “muskmelon-like” and “musky” flavours in cantaloupe [151].

**Figure 12:**
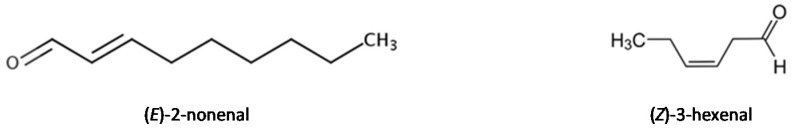
Chemical structure of (*E*)-2-nonenal and (*Z*)-3-hexenal.

Volatile aldehydes, also a chemical class formed by the lipoxygenase pathway from fatty acids [139,149], are well-known for their green note odour. (*E*)-2-nonenal and (*Z*)-3-hexenal (Figure 12), despite their different structures, also originate from different fatty acids ((*E*)-2-nonenal, from linoleic acid and (*Z*)-3-hexenal from linolenic acid) and are described as presenting other sensory attributes. “Penetrating”, “waxy” [150] or “fatty” [152] characteristics have been linked to (*E*)-2-nonenal, while, for (*Z*)-3-hexenal, “leafy”, “powerful”, “strawberry leaf”, “winey”, “green leaves”, “apple-like”, “leaf-like” and “cut grass” attributes have also been linked.

**Figure 13:**
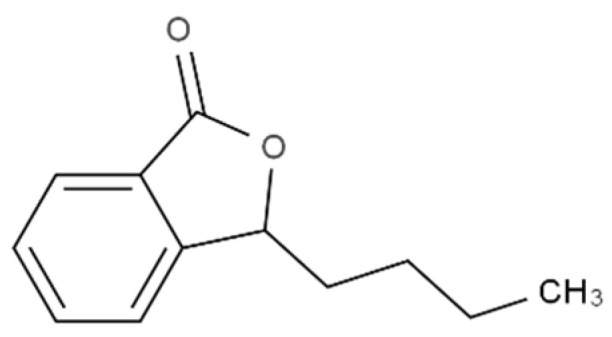
Chemical structure of 3-butylphthalide.

Sesquiterpene lactones are among the most prevalent and biologically significant classes of secondary metabolites found across the plant kingdom, comprising over 5000 known compounds, being most common in families such as *Cactaceae*, *Solanaceae*, *Araceae*, and the *Euphorbiaceae*. 3-Butylphthalide (Figure 13) is one of the most known lactones, and besides the “herbal” note associated with it, it is also mainly responsible for the “celery” aroma [153].

**Figure 14:**
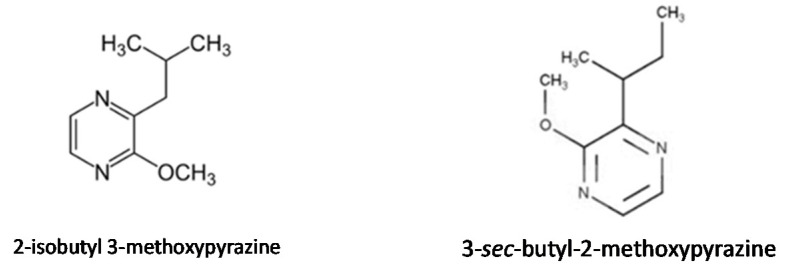
Chemical structure of 2-Isobutyl 3-methoxypyrazine and 3-*sec*-butyl-2-methoxypyrazine.

Pyrazines are heterocyclic compounds found in a wide variety of foods and are mostly associated with nutty and roasty flavours, as well as those of green vegetables. 2-Isobutyl 3-methoxypyrazine and 3-*Sec*-butyl-2-methoxypyrazine (Figure 14) are two well-known pyrazines that present low sensory detection thresholds, making them very important, as they can be the compounds responsible for the dominating aromatic features in several vegetables [154].

**Figure 15:**
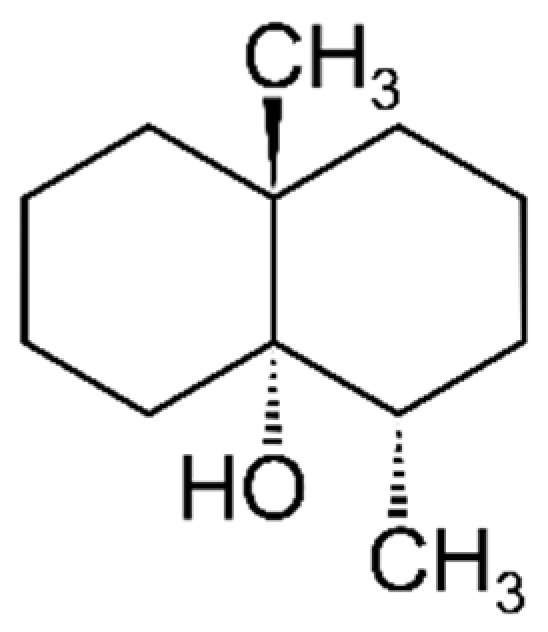
Chemical structure of geosmin.

The chemical class of terpenoids includes compounds widely distributed in plants and fruits and can be divided into two major groups: monoterpenes and sesquiterpenes or irregular terpenes, which are mostly synthesised in catabolic reactions and/or by autoxidation [155]. Geosmin (Figure 15) is an irregular terpene, and its major sensory attributes, as referred to, are “earthy” and “freshly plowed soil”.

**Figure 16:**
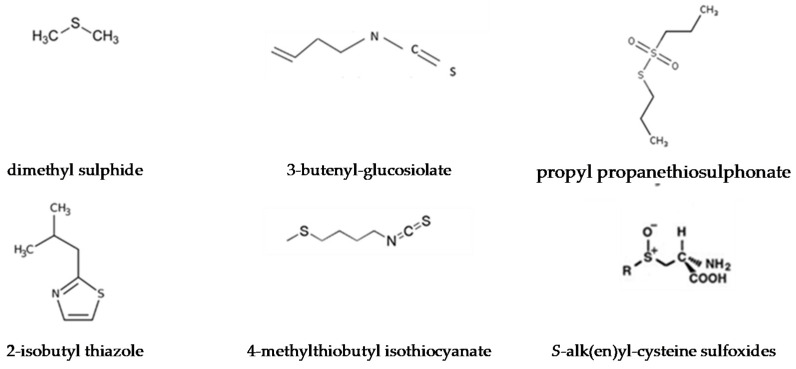
Chemical structure of sulphur-containing volatile compounds.

Sulphur-containing compounds (Figure 16) are synthesised from methionine and cysteine and can be emitted due to an increased accumulation of free methionine. They are key trace volatiles and are a major factor in the sensory properties of fruits and vegetables [156].

**Figure 17:**
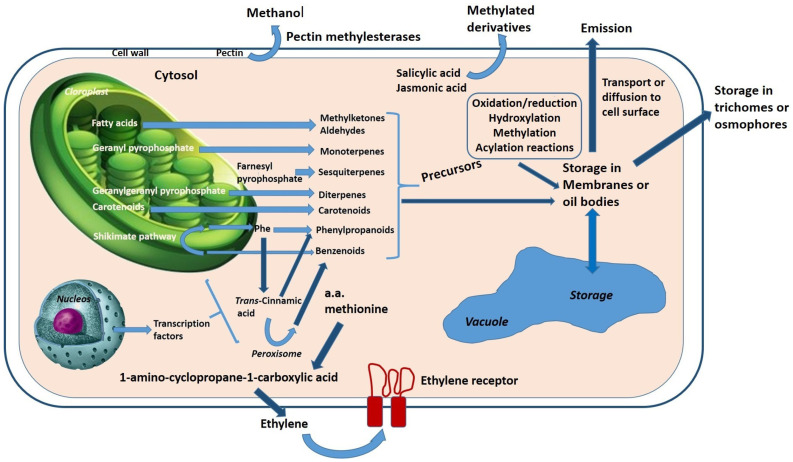
The principal plant volatile compounds are derived from four biosynthetic classes of precursors: terpenoids, fatty acid catabolites, aroma, and amino acid derived products. Many of these products are made more lipophilic (storage in membranes or oil bodies) before their release by removing or masking hydrophilic functional groups through reduction, methylation, or acylation reactions. Adapted from Baldwin et al. [157].

The formation of volatile and nonvolatile compounds is diverse, due to the multitude of molecules that convey flavour. In general, these molecules are synthesised from terpenoid, apocarotenoid, and lipoxygenase pathways and are derived from amino and fatty acids [139,149] (Figure 17).

**Figure 18:**
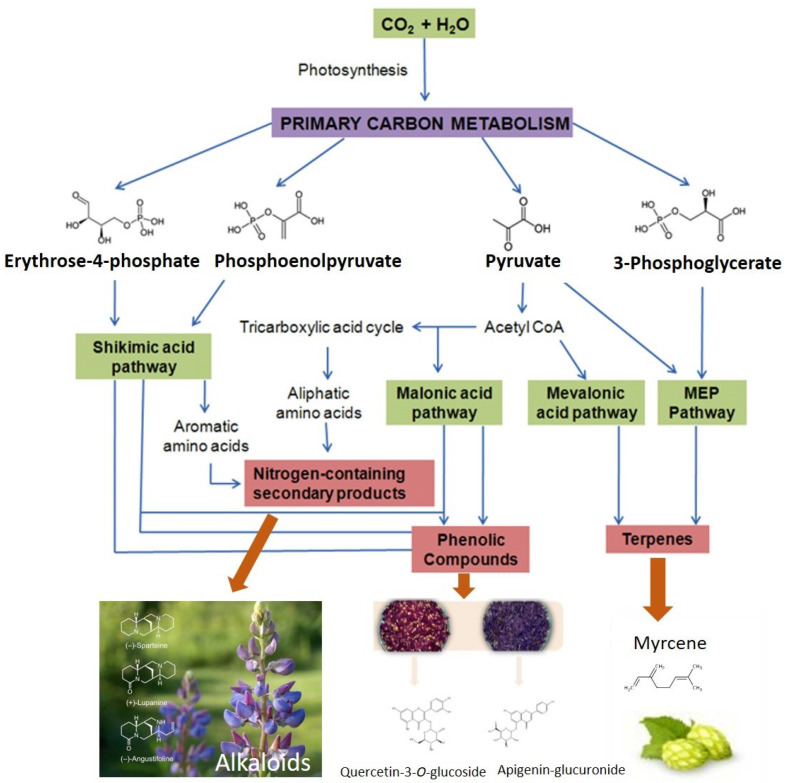
Generalized pathways for the synthesis of some nonvolatile compounds present in plants. Adapted from Ncube and Staden [161].

Although many of the volatile and nonvolatile compounds responsible for aromatic features have been identified, many of their biochemistry pathways are still not well explained. Still, several metabolic pathways are involved in the biosynthesis of compounds responsible for the aromatic features and taste in vegetables. Many volatile compounds are synthesised from fatty acid, amino acid, and carotenoid pathways [130,158], others from isoprenoid substrates. However, it is well-known that primary metabolism is fundamental for the formation of nonvolatile compounds, which also contribute to the aromatic features and taste of vegetables [158] (Figure 18). Among these are sugars, organic acids, free amino acids, provitamins, minerals, and salts [159]. For example, sweetness is determined by the concentrations of the predominant sugars, while sourness is determined by the concentrations of the predominant organic acids [160].

In tomato, its characteristic sweet-sour taste is due to a combination of the sugars and organic acids, and positive correlations between perceived sweetness, reducing sugar content, and soluble solids have been found [162]. Therefore, to define what compounds are more critical to flavour is more complex than expected. Moreover, when vegetables are harvested, a catabolic process starts due to the disruption of plant tissues, affecting their aroma and transforming their key flavours into different compounds. Some of them may even turn into a new biologically active compound. For example, in brassica vegetables, operations such as cutting, chewing, and cooking have an uncontrolled effect on volatile compounds, due to the mixture of enzymes. The brassica vegetables’ typical odours and tastes are mainly due to the presence of glucosinolates, which when in contact with the enzyme myrosinase (EC 3.2.1.147, thioglucoside glucohydrolase) hydrolyse into new groups of breakdown products such as isothiocyanates, organic cyanides, oxazolidinethiones, and thiocyanate [162], affecting their aroma and transforming their key flavour into a different one. A similar situation occurs with *Allium* species, such as onion, shallot, garlic, leek, and others, have in common the presence of the sulphur-based *S*-alk(en)ylcysteine sulfoxide (alliin, I) in their composition. In damaged or disrupted tissue transformation into several other compounds, via alliinase occurred [163]. The initial hydrolysis products are ammonia, pyruvate, and an alk(en)ylthiosulphinate (allicin, II), and can undergo further nonenzymatic reactions to yield a variety of compounds such as thiosulphate [III] and di- and trisulphides [IV] [146], which gives to *Allium* species their typical odour of a sulphur-like smell.

Although most of such compounds are related to odour and flavour, some of them are also involved in important biochemical activities. Epidemiological studies have shown that glucosinolate hydrolysis products (responsible for the bitterness and mustard/horseradish-like flavours) may act as an anticarcinogen agents and can exert antibacterial and antifungal activities against diverse human and plant pathogens [164]. Recently, allicin, a derived compound from alk(en)ylcysteine sulfoxide in *Allium* species, showed antimicrobial and anticarcinogenic activities [165,166]. Compounds such as lycopene and carotene, largely present in tomato, carrot, and spinach, have been associated with anti-inflammatory properties [167].

### 3.2. MAP Volatile Compounds Responsible for Aromatic Features

Plants provide multiple ranges of aromatic features well-noticed by the most sensitive human senses—taste, and odour [168]. Over time, many plant species have been used to produce foods and medical or herbal formulations [169]. The use of MAPs began as an unselective wild-harvesting of plants, moving into a selective collection and then to the cultivation of the most useful. From ancient times to the present day, plants have been used as medicines and food preservers [170]. Nowadays, their cultivation, pharmacognosy, phytochemistry, biology, conservation, and sustainable use are matters of interest [171]. MAPs yield a wide variety of natural compounds, produced and stored in glands located in different parts of the plant: leaves, flowers, fruits, seeds, barks, and roots [172,173]. These natural compounds, most of which are essential oils, are volatile at room temperature, and important for plant adaptation and survival—namely as pollinator attractants, as herbivores restraints, or as a defence against pathogenic microorganisms. Because of their biological activities, they are also important to Man in both commercial and industrial resources—namely, in traditional medicine [172], which also provides raw materials for use in pharmaceuticals, cosmetics, food, and chemical industries [173].

What are the MAPs? According to Maiti and Geetha [174], MAPs are plants that provide “medicines” to humans that prevent disease, maintain health, or cure illnesses; “let food be your medicine”, attributed to Hippocrates, 460–377 B.C., is again a popular concept. New designations have emerged to classify the beneficial effects of the use of some plants or plant parts and products. According to Barata et al. [172], MAPs can be divided into four groups, based on their final usage: raw materials for essential oil extraction, which is the major use of MAPs around the world; spices, nonleafy parts of plants used as flavouring or seasoning; herbs, leafy or soft flowering parts of the plant used as flavouring or seasoning; miscellaneous group, MAPs used in different ways.

The International Union for Conservation of Nature and the World Wildlife Fund estimated that about 50,000–80,000 flowering plant species are used in medicinal formulations across the world. Among these, only 1 to 10% has been studied chemically and pharmacologically for their potential value [175]. MAPs contain a wide variety of bioactive secondary metabolites, such as essential oils, alkaloids, phenolics (such as flavonoids), steroids, terpenes, sesquiterpenes, diterpenes, and saponins [176], that find uses in several perfumeries, flavourings, and pharmaceutical compounds [177]. Many secondary metabolites include aroma substances, and phenolic compounds or their oxygen-substituted derivatives such as tannins [178], and many of these compounds have anti-inflammatory and antioxidant properties. Plant secondary metabolites are characterised as exhibiting chemical polymorphism, which causes the occurrence of several chemotypes within the same species [179]. The chemotypes are of extreme importance when considering the safety, quality, and efficacy of herbal products derived from MAPs. There are numerous cases of plant species showing a great variety of chemotypes. The genus *Thymus* shows many examples since many *Thymus* species are chemically heterogeneous. *Thymus vulgaris* is among the most popular plants having chemotypes. Six different chemotypes are known, depending on the main component of the essential oil: thymol, carvacrol, linalool, geraniol, borneol, sabinete hydrate, and multiple component chemotypes [180].

Table 6 summarises some important MAPs and their main volatile compounds. Regarding the data presented in this table, a different type of compound is responsible for the aromatic features of MAPs. For example, Lee et al. [181] identify a high content of linalool, methyl cinnamate, estragole, eugenol, and 1,8-cineole compounds in basil cultivars and reported that these compounds were responsible for the typical aroma of basil perceived by consumers. Similar results were presented by Shahwar et al. [182], who reported that the typical aroma of coriander is due to a mixture of different compounds in which decenal and related compounds (Table 6) assume a high preponderance. Several other authors [181,182,183,184,185,186,187,188,189,190,191] have reported that the typical aromatic features exhibited by MAPs are a result of a combined effect of several compounds rather than a single compound, as shown by Kizhakkayil and Sasikumar [185] for ginger. These authors reported that the typical “spicy” and “fresh” aromas exhibited by ginger is due to the simultaneous presence of different compounds such as zingiberene, 6-gingerol, 8-gingerol, 10-gingerol, 6-shogaol, 8-shogaol, geranial, and neral. These compounds, even in lower amounts, are critical for the consumer to perceive the typical aroma of ginger, all of them are important to define the ginger “bouquet”.

For better exploitation of any MAP species, it is necessary to evaluate the genetic stability of their populations, i.e., whether such populations continue to produce the same characteristic products after being transplanted to and grown in habitats with different edaphoclimatic conditions. In all, for each species, it is crucial to perform a detailed study, which addresses, for instance, the influence of seasonal and geographic variations and local environmental conditions. The results obtained will provide the scientific basis for the selection and cultivation of species showing better qualities, thus bringing some economic and social benefits for local growers.

### 3.3. Advances in Aromatic Features and Future Research Perspectives

From the aromatic features perspective, the actual challenges in research are multifold: (a) overcome the crop defects; (b) refinement of aroma deviations; (c) modulate volatile and nonvolatile compounds’ biosyntheses to produce high-potency aromatic features; (d) increase the accuracy of aromatic features signature; (e) understand how preharvest and postharvest factors can affect vegetable and MAP aromatic features or tastes. The flavour is the result of a complex metabolic network that can be influenced by several factors, such as genetics, environment, agricultural practices, and postharvest handling and storage. However, recent findings show that the biosynthesis of the compounds can be remarkably influenced by other factors, such as enzyme specificity of gene adaptation [192,193] leading to the research of new steps. Until recently, the research focus was to understand how agriculture practices affect plant composition and interfere in the consumer´s perception of aromatic features [126,194], but the latest research studies are shifting from yield to quality factors. Nowadays, the trends in consumption are mostly defined by consumer’s preferences (sustainability, nutrition, aromatic features, novelty) and not exclusively by the producer’s priorities [194]. Genomic and metabolomic analysis with clarification of the fundamental metabolism of volatile compounds with aromatic features and their biosynthetic mechanisms, regulations, and localisation is a hot topic [194]. So, linkages of the biosynthesis of aromatic features with enzymatic endogenous processes will provide new insights into the flavour control mechanism. Moreover, the association of genome and metabolome analysis with identification of key enzymatic changes occurred in physiological processes would address new opportunities to increase the contents of specific compounds, particularly those with importance for consumer acceptance. This approach will open the possibility to produce vegetables and MAP species with an enhanced content of a specific volatile or nonvolatile compound, with greater biological properties. Additionally, it will speed up the discovery of new or unknown chemosensory-active molecules and understanding of their biochemical interactions with main food matrix constituents. Likewise, it will open ways of direct improvement of foods by adapting processing parameters that can help to overcome taste defects or undesirable aromatic features, without the addition of any artificial ingredients.

## 4. Final Remarks

Vegetables and MAPs are two important sources of bioactive and volatile compounds that are responsible for consumer perceptions on their importance in human health. Hundreds of studies using in vitro and in vivo models have shown that phenolics and volatile compounds are directly involved in different degenerative cellular mechanisms and thus are being considered as key compounds to reduce or to inhibit pro-oxidant and inflammatory processes. The combination of such compounds gives us an important view on the quality of vegetables and MAPs. Thus, it is important to understand what types of compounds are present in vegetables and MAPs, the relations between them, and how they can be affected by biotic or abiotic factors. This review summarises all these aspects. This information is important to a better understanding of all the processes behind the formation of volatile and nonvolatile compounds as well as their bioactivities, their interaction with other compounds, but, more importantly, how they influence the consumer’s perception of quality. Moreover, their influence on the human tendency to buy vegetables and MAPs is also supported by this type of information. This is true to all plant species reported in this work, but also to those not included here, and a continuous effort to identify volatile and nonvolatile compounds is ongoing. Furthermore, the improvement of aromatic features is fundamental and must be achieved without compromising other quality traits of crops.

## Figures and Tables

**Table 1:** Phenolic compounds in *Salvia officinalis*, *Thymus vulgaris*, and *Rosmarinus officinalis* (mg/100 g of fresh weight). Data from Zheng and Wang [22].

Phenolic Compounds	*Salvia officinalis*	*Thymus vulgaris*	*Rosmarinus officinalis*
Vanillic acid	2.27 ± 0.48		1.73 ± 0.08
Caffeic acid	7.42 ± 0.35	11.7 ± 1.04	2.95 ± 0.12
Luteolin	33.4 ± 1.32	39.5 ± 1.53	
Rosmarinic acid	117.8 ± 1.01	91.8 ± 2.75	32.8 ± 1.69
Hispidulin	16.3 ± 1.07	20.8 ± 0.96	19.7 ± 1.12
Cirsimaritin	14.3 ± 0.83		24.4 ± 0.87
Carnosic acid			126.6 ± 6.00
Apigenin	2.4 ± 0.07		1.1 ± 0.15
Naringin			53.1 ± 2.09
Rosmanol			124.1 ± 3.19
Total phenolic (mg of GAE/g of fresh weight)	1.34 ± 0.09	2.13 ± 0.11	2.19 ± 0.15
ORAC (Oxygen Radical Absorbance Capacity—µmol of TE/g of fresh weight)	13.28 ± 0.40	19.49 ± 0.21	19.15 ± 0.63

**Table 2:** Phenolic compounds present in some vegetables consumed worldwide and the main bioactivities pointed.

Vegetables	Main Phenolics	Bioactivities Pointed	Ref.
Artichoke (*Cynara scolymus* L.)	Hydroxytyrosol, verbascoside, apigenin-7-glucoside, oleuropein, quercetin, pinoresinol, and apigenin	Anti-inflammatory activities of *C. scolymus* were found due to the synergistic effect of phenolic compounds. Inhibitory action of artichoke extracts in the inflammatory process such as histamine, bradykinin, and chemokine mediators’ processes was related to the phenolic content.	[78]
Broccoli florets (*Brassica oleracea* L. var. *italica*)	Hydroxybenzoic acid, hydroxycinnamic acid, flavone, polymethoxylated flavone, kaempferol glycosylated and kaempferol derivatives, quercetin-3-*O*-glucoside and derivatives, isorhamnetin-3-*O*-rutinoside, isorhamnetin glucoside, and related compounds	Hydroalcoholic extracts were capable of directly reacting with and quenching DPPH and Oxygen (ORAC) radicals. Flavonoids and derivatives showed significant positive correlations to DPPH, and ORAC.	[82]
Celery (*Apium graveolens* L.)	High content of apiin, apigenin, and rutin, 3,7-dihydroxyflavone, cyanidin and diosmetin, and terpenes (α-ionone)	Antioxidant activity was highly correlated with the presence of apiin, apigenin, and rutin, mainly due to the lower BDE of O–H bonds in their B rings, which enhanced their H atom donating ability.	[83]
Garlic (*Allium sativum* L.)	The high content of total phenolic content, vanillic acid, caffeic acid, *p*-coumaric acid, ferulic acid, sinapic acid, cyanidin-3-(6′-malonyl)-glucoside)	A positive and significant correlation between the content of total phenolic content and antimicrobial and antioxidant activity was found. The highest total phenolics content was significantly correlated with the lowest EC50 values for all the tested antioxidant activity assays.	[84,85]
Ginseng leaves (*Panax ginseng* C. A. Mey.)	Gallic acid and galangin	The antioxidant capacity in the lipophilic fraction was higher than those in hydrophilic fractions and positive correlations between antioxidant capacity and total phenolic content, gallic acid, and galangin were found.	[86]
Leek (*Allium porrum* L.)	Rosmarinic acid, quercetin, and apigenin glycosylated forms and respective derivatives	Extracts showed a favourable antimicrobial activity against *Staphylococcus aureus*, *Bacillus subtilis*, and *Aspergillus niger*. Extracts inhibit Hep2c, L2OB, and RD tumor cells in a dose-dependent manner after 48 h treatment period.	[87]
Onion (*Allium cepa* L.)	Quercetin aglycone, quercetin-4′-*O*-monoglucoside, and quercetin-3,4′-*O*-diglucoside	The antioxidant activity of onions was dependent on variation in the contents of quercetin compounds in all onion varieties assessed. Antibacterial activity against *Staphylococcus* sp. and *Escherichia coli* was dependent on variation in both phenolic profile and content.	[79]
Watercress (*Nasturtium officinale* L.)	Coumaric acid, sinapic acid, caftaric acid, quercetin, and quercetin derivatives were the major phenolic compounds identified	The radical scavenging activity (RSA) of root, stem, and leaves of watercress methanolic extracts were highly correlated with the variation of phenolics. Watercress leaves had similar antioxidant potential to that of tocopherol.	[88]

**Table 3:** The key role of some important phenolics identified in some medicinal and aromatic plant (MAP) species extracts and respective bioactivities.

MAP Extracts	Main Phenolics Identified	Bioactivities Pointed	Ref.
Fern (*Asplenium nidus* L.)	7-*O*-hexoside and quercetin-7-*O*-rutinoside	Antimicrobial activity against *Proteus mirabilis* Hauser, *Proteus vulgaris* Hauser, and *Pseudomonas aeruginosa* (Schroeter). Migula was shown when fern extracts were applied at different concentrations.	[89]
Ginkgo leaves (*Ginkgo biloba* L.)	Quercitin-3-*O*-glucoside	Ginkgo leaf extracts were capable of decreasing sunburn symptoms in UVB-induced skin in vivo models.	[90]
Green tea (*Camellia fangchengensis* Liang and Zhong)	Procyanidin B1, B2, B3, procyanidin trimer, fangchengbisflavan A and B, catechin 7-*O*-β-glucopyranoside, epicatechin, (−)-epicatechin gallate, epigallocatechin, and epicatechin 3-(3-*O*-methyl) gallate	Antiradical and antioxidant activity against in vitro studies was shown.	[91]
Haskap berry (*Lonicera caerulea* L.)	Cyanidin-3-*O*-glucoside, cyanidin-3-*O*-rutinoside, chlorogenic acid, quercitin-3-*O*-rutinoside, quercitin-3-*O*-glucoside, and catechin	Extracts exhibited comparable anti-inflammatory effects to diclofenac which is a COX inhibitory medicine.	[92]
Nutmeg (*Myristica fragrans* Houtt)	30,40,7-trihydroxyflavone	Antibacterial activity of nutmeg extracts against the multidrug resistant Gram-negative bacteria *Providencia stuartii* Ewing and *Escherichia coli* was observed.	[80]
Lavandula (*Lavandula pedunculata* Mill.)	Caffeic acid, luteolin-7-*O*-glucuronide, and rosmarinic acid	Exhibited highest anti-inflammatory activity in rat RAW 264.7 macrophages by inhibiting nitric oxide production.	[93]
Rosemary (*Rosmarinus officinalis* L.)	Isorhamnetin-3-*O*-hexoside, carnosic acid, carnosol, rosmanol, epirosmanol, rosmaridiphenol, rosmarinic acid, and their methoxy derivatives	Antioxidant and antiradical activities were observed. Exerted a direct cytocidal effect via upregulation of nitric oxide (NO) in cancer cells, which in turn acts in a proapoptotic manner and induces cell apoptosis.	[94]
Oregano (*Origanum vulgare* L.)	Rosmarinic acid, 3,4-dihydroxybenzoic acid	The hydroalcoholic extract shows antioxidant activity in vitro and in vivo models. The oral formulation of oregano preserves antioxidant activity from gastrointestinal digestion.	[81]
Thymus (*Thymus algeriensis* Boiss. and Reut)	Rosmarinic acid, caffeoyl rosmarinic acid, eriodictyol hexoside, kaempferol-*O*-hexoside, kaempferol-*O*-hexuronide, luteolin-*O*-hexuronide, apigenin-C-di-hexoside, and apigenin-*O*-hexuronide	Methanolic extracts were found to possess substantial antioxidant and antiacetylcholinesterase activities which were correlated to their phenolic contents; however, significant variations were observed between populations.	[95]
Sage (*Salvia officinalis* L.)	Apigenin, carnosic acid, carnosol, rosmanol, epirosmanol, rosmarinic acid, and their methoxy derivatives	Antioxidant and antiradical activities were observed. Sage extracts were capable of exerting a direct cytocidal effect via upregulation of nitric oxide (NO) in cancer cells, in a proapoptotic manner which induced cell apoptosis.	[94]

**Table 4:** Key volatile and nonvolatile compounds present in some vegetables largely consumed worldwide. The sensory attributes were adapted from Parker et al. [126] and Maarse [134].

Vegetables	Key-Volatile Compounds	Sensory Attributes	Ref.
Broccoli (*Brassica oleracea* L. var. *italica*)	Methanethiol, hydrogen sulphide, dimethyl disulphide, trimethyl disulphide, dimethyl sulphide, hexanal, (*Z*)-3-hexen-1-ol, nonanal, ethanol, 4-methylthiobutyl isothiocyanate, butyl isothiocyanate, 2-methyl butyl isothiocyanate, and 3-isopropyl-2-methoxypyrazine	“Cabbage”, “radish”	[135]
Cabbage (*Brassica oleracea* L. var. *capitate*)	2-Propenyl isothiocyanate, methanethiol, dimethyl sulphide, dimethyl trisulphide, ethanol, methyl acetate, ethyl acetate, hexanal, (*E*)-2-hexenal, and (*Z*)-3-hexen-1-ol	“Sulphury”, “onion”, “sweet corn”	[136,137]
Cauliflower (*Brassica oleracea* L. var. *botrytis*)	2-Propenyl isothiocyanate, dimethyl trisulphide, dimethyl sulphide, and methanethiol	“Sulphur”, “cauliflower”, “putrid”	[138,139]
Carrot (*Daucus carota* L. subsp. s*ativus*)	α-Pinene, sabinene, myrcene, limonene, β-ocimene, γ-terpinene, *p*-cymene, terpinolene, β-caryophyllene, α-humulone, (*E*)-γ-bisabolene and β-ionone, 3-*sec*-butyl-2-methoxypyrazine	“Earthy”, “fruity”, “citrus-like”, “woody”, and “sweet”	[134]
Celery (*Apium graveolens* L.)	3-Butylphthalide and 3-butyltetrahydrophthalide (sedanolide), (*Z*)-3-hexen-1-ol, myrcene, limonene, α-pinene, γ-terpinene, 1,4-cyclohexadiene, 1,5,5-trimethyl-6-methylene-cyclohexene, 3,7,11,15-tetramethyl-2-hexadecen-1-ol, and α-humulene	“Herbal”	[140,141]
Cucumber (*Cucumis sativus* L.)	3-Isopropyl-2-methoxypyrazine, (*E, Z*)-2,6-nonadienal, and (*E*)-2-nonenal	“Fatty”, “green”, “cucumber”	[140,142]
Garlic (*Allium sativum* L.)	Allicin, *S*-alk(en)yl-cysteine sulfoxides, di-2-propenyl disulphide, methyl 2-propenyl disulphide, dimethyl trisulphide, methyl 2-propenyl trisulphide, and di-2-propenyl trisulphide	“Ammonia”, “sulphur-like smell”	[143]
Leek (*Allium porrum* L.)	1-Propanethiol, dipropyl disulphide, dipropyl trisulphide, methyl(*E*)-propenyl disulphide, and propyl (*E*)-propenyl disulphide	“Onion”, “green”	[144,145]
Onion (*Allium cepa* L.)	*S*-alk(en)yl-cysteine sulfoxides, thiopropanal-*S*-oxide (the lachrymatory factor) 3,4-dimethyl-2,5-dioxo-2,5-dihydrothiophene, propyl methanethiosulfonate, and propyl propanethiosulfonate	“Ammonia”, “sulphur-like smell”	[144]
Pea (*Pisum sativum* L.)	Hexanal, (*E*)-2-heptenal, (*E*)-2-octenal, 1-hexanol, (*Z*)-3-hexen-1-ol, 3-alkyl-2-methoxypyrazines, 3-isopropyl-2-methoxypyrazine, 3-*sec*-butyl-2-methoxypyrazine, 3-isobutyl-2-methoxypyrazine, 5-methyl-3-isopropyl-2-methoxypyrazine, and 6-methyl-3-isopropyl-2-methoxypyrazine	“Green”, “herbal”	[146]
Pepper (*Capsicum annuum* L.)	(*Z*)-3-hexenal, 2-heptanone, (*Z*)-2-hexenal, (*E*)-2-hexenal, hexanol, (*Z*)-3-hexanol, (*E*)-2-hexenol, and linalool, 2-Isobutyl 3-methoxypyrazine	“Green pea”, “green bell pepper”, “spicy”, “herbal”	[133]
Tomato (*Solanum lycopersicum* L.)	Hexanal, cis -3-hexenal and *trans* -2-hexenal, hexanol, cis -3-hexenol, 1-penten-3-one, 2-isobutylthiazole, 6-methyl-5-hepten-2-one, β-ionone, 3-methylbutanal, 3-methyl butanol, 2-pentenal, acetone, ethanol and fureanol, (*Z*)-3-hexenal	“Green”, “wasabi”, “privet”, “tomato leaf”, “fatty”, “grassy”	[147,148]

**Table 5:** The key role of some volatile compounds responsible for the sensory attributes of some vegetable species adapted from Parker et al. [126] and Maarse [134].

Vegetables	Volatile Compound	Sensory Attributes
	Alcohols	
Watermelon	(*Z*, *Z*)-3,6-Nonadienol	“Fatty”, “soapy”, “cucumber”, “watermelon”, “rind”
	Aldehydes	
Cucumber	(*E*)-2-nonenal	“Fatty”, “green”, “cucumber”
Tomato	(*Z*)-3-hexenal	“Green”, “fatty”, “grassy”
	Lactones	
Celery	3-Butylphthalide	“Herbal”
	Pyrazines	
Green bell pepper, peas	2-Isobutyl 3-methoxypyrazine	“Green pea”, “green bell pepper”, “spicy”, “herbal”
Carrot	3-*sec*-butyl-2-methoxypyrazine	“Earthy”, “fruity”, “citrus-like”, “spicy”, “woody”, and “sweet”
	Terpenoids	
Red beet	Geosmin	“Freshly plowed soil”, “earthy”
	Sulphur compounds	
Asparagus, cabbage	Dimethyl sulphide	“Sulphury”, “onion”, “sweet corn”
Tomato	2-Isobutyl thiazole	“Green”, “wasabi”, “privet”, “tomato leaf”
Turnip	3-Butenyl-glucosinolate	“Bitter taste and aftertaste”
Broccoli	4-Methylthiobutyl isothiocyanate	“Cabbage”, “radish”
Onion	Propyl propanethiosulfonate	“Roasted alliaceous”
Radish	4-Methylthio-3-butenyl-isothiocyanate	“Sharp taste”, “mustard/horseradish-like”
Garlic, onion	*S*-alk(en)yl-cysteine sulfoxides	“Ammonia”, “sulphur-like smell”

**Table 6:** Key volatile compounds present in some medicinal and aromatic plants (MAPs). In bold are the major volatile compounds of each MAP. The names of the compounds in bold are those represented in the figures.

MAP	Main Volatile Compounds	Chemical Structure of Major Volatile Compounds	Ref.
Basil (*Ocimum basilicum* L.)	**Linalool**, methyl cinnamate, estragole, eugenol, and 1,8-cineole	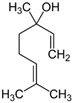	[181]
Coriander (*Coriandrum sativum* L.)	**(*E*)-2-Decenal**, linalool, (*E*)-2-dodecenal, (*E*)-2-tetradecenal, 2-decen-1-ol, (*E*)-2-undecenal, dodecanal, (*E*)-2-tridecenal, (*E*)-2-hexadecenal, pentadecenal, and α-pinene	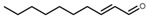	[182]
Fennel (*Foeniculum vulgare* (Mill.)	***trans*-Anethole**, estragole, fenchone, and 1-octen-3-ol	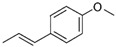	[183]
Ginger (*Zingiber officinale* Rosc.)	**Zingiberene**, 6-gingerol, 8-gingerol, 10-gingerol, 6-shogaol, 8-shogaol, 10-shogaol, geranial, neral, 1,8-cineole, β-bisabolene, β-sesquiphellandrene, (*E*)(*E*)-α-farnesene, viridiflorol, and (*E*)(*E*)-farnesal	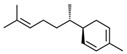	[184,185]
Lavender (*Lavandula angustifolia* Mill.)	**1,8-Cineole**, camphor and borneol	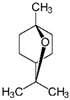	[186]
Melissa (*Melissa officinalis* L.)	**Geranial**, neral, alloaromadendrene, geranyl acetate, 6-methyl-5-hepten-2-one, and β-caryophyllene	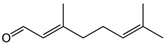	[187]
Oregano (*Origanum vulgare* L.)	**Sabinene**, 1,8-cineole, caryophyllene oxide, (*E*)-β-caryophyllene, *p*-cymene, α-terpineol, and germacrene D	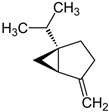	[188]
Parsley (*Petroselinum crispum* (Mill.) Nym. Ex A.W.Hill	**α-Pinene**, sabinene, myrcene, β-pinene, *cis*-3-hexenyl acetate, α-phellandrene, *p*-cymene, limonene, β-phellandrene, *trans*-β-ocimene, γ-terpinene, terpinolene, 1,3,8-p-menthatriene, α-terpineol, *trans*-β-caryophylle, germacrene-D, nerolidol, and myristcin	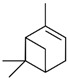	[189]
Peppermint (*Mentha* x *piperita* L.)	**Santene**, camphene, β-pinene, myrcene, *cis*-3-hexenyl acetate, *p*-cymene, α-terpinene, limonene, *trans*-β-ocimene, γ-terpinene, *trans*-sabinene hydrate, nonanal, linalool, *cis*-limonene oxide, *trans*-limonene oxide, and *cis*-*p*-mentha-2,8-dien-1-ol	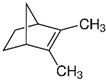	[189]
Rosemary (*Rosmarinus officinalis* L.)	**α-Pinene**, myrcene, 1,8 cineole, camphor, caryophyllene, α-humulene, nerolidol, spathulenol, and rosmarinic acid	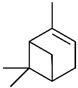	[190]
Thymus (*Thymus vulgaris* L.)	**Thymol**, carvacrol, linalool, geraniol, borneol, and sabinete hydrate	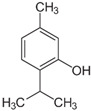	[180]
Sage (*Salvia officinalis* L.)	**α-Thujone**, 1,8 cineole, β-caryophyllene, α-humulene, α-pinene, β-thujone, β-pinene, camphene, camphor, and *p*-cymene	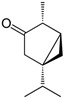	[191]

## Data Availability

Not applicable.
